# Development of a New Type of Prolonged Release Hydrocodone Formulation Based on Egalet® ADPREM Technology Using *In Vivo*–*In Vitro* Correlation

**DOI:** 10.3390/pharmaceutics3010073

**Published:** 2011-03-09

**Authors:** Pernille H. Hemmingsen, Anne-Mette Haahr, Christine Gunnergaard, Jean-Michel Cardot

**Affiliations:** 1 Egalet Denmark, Lejrvej 37-41, DK-3500 Værløse, Denmark; 2 Biopharmaceutical Department, ERT-CIDAM, Faculty of Pharmacy, University of Auvergne, 28 Place H. Dunant, BP 38, 63001 Clermont-Ferrand, France

**Keywords:** hydrocodone, controlled release, opioid, pain management, IVIVC, dissolution, Abuse Deterrent Erodible Matrix technology, oral solid dosage form

## Abstract

A novel abuse deterrent, prolonged release tablet formulation of Hydrocodone for once-daily dosing has been developed, based on the novel proprietary Egalet® ADPREM technology. The tablet is an injection molded polymer system consisting of an erodible matrix in which the Active Pharmaceutical Ingredient (API), such as Hydrocodone, is dispersed. The matrix is partly covered with a water-impermeable, non-erodible shell which leaves both ends of the cylindrical tablet exposed to erosion by the gastrointestinal (GI) fluid. *In vivo*–*in vitro* correlation (IVIVC) was initiated and validated with three formulations. A good internal predictability was observed for the three formulations. How the changing conditions in the GI tract influenced *in vivo* performance of an erosion based product was discussed. The validated IVIVC could be used to optimize the tablet formulation and to obtain a desired profile. In addition, this technique could help to establish the dissolution limits in which a certainty of bioequivalence is calculated. Based on this validated level A IVIVC, dissolution can be used as surrogate of bioequivalence for development, but also scale up post approval changes.

## 1. Introduction

Historically, Hydrocodone has been used primarily as cough medicine [1,2] and for treating acute post operative pain [3]. Over time, there has been an increasing need for usage of the drug in relation to chronic pain in, among others, cancer, persistent musculoskeletal pain such as osteoarthritis and lower back pain [4].

In recent years, Hydrocodone has become the most commonly prescribed opioid and is available in over 200 different products in the United States. Hydrocodone is prescribed as either antitussive or analgesic for treating moderate to moderately severe pain [5].

Pharmacodynamically Hydrocodone resembles codeine and morphine, being approximately equipotent to the latter [6].

Oral Hydrocodone is known from marketed combination products containing low doses of 5 to 10 mg Hydrocodone co-formulated with acetaminophen or ibuprofen. These products are, however, immediate release (IR) products which may be taken every three to six hours. In a clinical setting, this is not optimal for around the clock pain therapy.

A novel abuse deterrent, prolonged release tablet of Hydrocodone for once-daily dosing has been developed based on the novel proprietary Egalet® ADPREM technology (Abuse Deterrent Prolonged Release Erodible Matrix technology). The formulation is an injection molded polymer system consisting of an erodible matrix in which Active Pharmaceutical Ingredient (API) like Hydrocodone is dispersed. The matrix is partly covered with a water-impermeable, non-erodible shell which leaves both ends of the cylindrical tablet exposed to erosion by the gastrointestinal (GI) fluid. The tablet strength is defined by the initial API loaded into the tablet. The release rate is controlled by the well-defined, fixed size of the surface erosion area at both ends of the tablet. This allows a tightly controlled, prolonged release of the API which is only limited by the time of residence in the GI tract, the tablet geometry and composition. In addition, the tablet is designed to maintain its prolonged-release properties across a wide range of solvents, and is resistant to physical and chemical attempts to alter the slow-release of the API contained in the tablet.

*In vitro*–*in vivo* correlation (IVIVC) can be used to shorten development duration in respect of the safety and overall quality of the product. IVIVC can be used: (i) to predict human absorption and then *in vivo* plasma concentration profiles based on *in vitro* data; (ii) to optimize dosage forms with the fewest possible trials in man; (iii) to set biorelevant dissolution acceptance criteria; and (iv) as a surrogate for further bioequivalence studies in the frame of biowaivers for post approval variations. This correlation is possible as dissolution reflects the global performance of the product and all the phenomena underlined such as: formulation, process, excipients, ageing, and API characteristics [7,8]. In the case of a highly soluble and permeable API, such as Hydrocodone, and a prolonged release tablet, a difference in dissolution is more likely due to a difference in the tablet composition or process that will impact the global in vivo performance.

The purpose of the current paper is to present how IVIVC were used: (i) to develop and optimize a new Hydrocodone Prolonged Release (PR) tablet based on the novel ADPREM technology; and (ii) to fix limits of dissolution in order to be bioequivalent between the tablets and batches during scale up, but also in case of post approval change [9-12] and thus to either minimize the risk of *in vivo* studies or to be used as a surrogate of bioequivalence.

## 2. Experimental Section

### 2.1. Tablet formulation

Hydrocodone exhibits a high solubility in aqueous solvents and a good permeability. It is extensively metabolized in liver by CYP2D6 into Hydromorphone, a more potent opioid.

**Figure 1.**
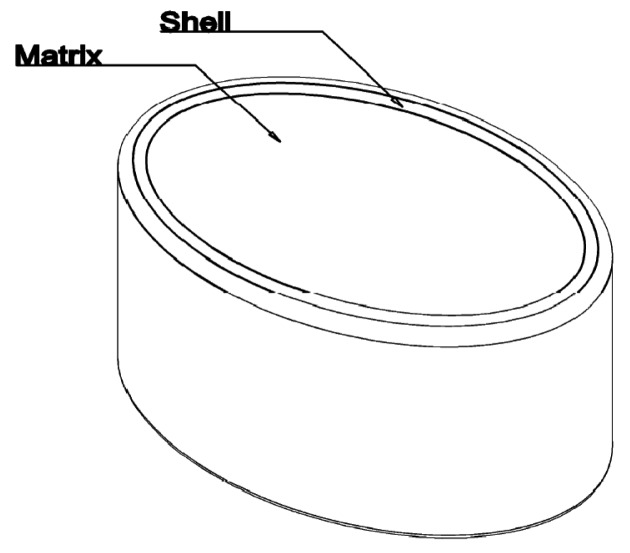
Egalet® ADPREM tablets.

Three tablets (A to C) were developed based on Egalet® technology, all of them containing 20 mg of Hydrocodone as tartrate salt. The three tablets are based on one common qualitative and quantitative composition of excipients and a fix mass of Hydrocodone per tablet. They differ solely by the mass of the final tablet, corresponding to different diameters and lengths of the tablet (6, 7.5 or 9 mm, respectively) and adjusted by an increase of excipients' mass. The main characteristics of the tablets are presented in Table 1 and Figure 1.

The tablet batches were prepared according to Good Manufacturing Practice (GMP) by two component injection molding, employing an Arburg Allrounder 420 V 800-60/35 injection molding machine (Arburg, Greve, Denmark). The geometrical shape of the formulations was defined by a custom made stainless steel mold (ATZ form, Copenhagen, Denmark).

An IR tablet containing 10 mg of Hydrocodone in combination with 325 mg of Paracetamol (NORCO® 10/325 immediate-release tablet (Watson Pharma, lot 102663A) was also included in the clinical study as a reference and in order to perform deconvolution.

### 2.2. Dissolution studies

The dissolution profiles of the three test tablets were realized using a Pharmacopoeia media: phosphate buffer pH 6.8, using a USP Apparatus 2 paddle method (Vankel VK7025 coupled to a Varian Cary 50 UV-visible spectrophotometer). The dissolution volume was 1,000 mL. The paddle speed and temperature were set at 50 rpm and 37 °C, respectively. Samples were withdrawn at predefined time intervals (15 min) up to 600 minutes in a close loop system and were directly filtered with a 70 μm probe filter and subsequently measured by UV on line detection.

### 2.3. *In vivo* bioavailability assessment

A pharmacokinetic (PK) study based on a 4-arm, single dose, randomized cross-over design comparing the three test tablets to the reference formulations, NORCO®, was performed on 25 fasted subjects. The PK plasma samples were collected at predefined time intervals from 0 to 42 hours and measured by a validated HPLC-MS method. Classical bioavailability parameters were calculated: Maximum observed concentration and time to obtain it (Cmax and Tmax) and extent absorbed: area under the curve (AUC). As mentioned in the guidelines [12], bioequivalence parameters under consideration were Cmax and AUC. In case of prolonged release formulation, Tmax is of minor relevance and was not analyzed.

The absorption kinetics were calculated using a deconvolution technique using the IR reference tablet as response function according to the method described by Langenbucher [13-15].

The deconvolution technique was applied independently of any modelization of the absorption. Deconvolution allows isolating the input (« absorption ») function by a numerical algorithm as a function of the observed concentration for the studied tablet and for the IR reference tablet. In the current case this input function reflected the in vivo release observed after administration of the PR test tablets. The simulations of the curves from the theoretic input were performed using convolution [13-15].

### 2.4. IVIVC

The clinical study was designed to support a level A correlation. Level A correlation is “a point-to-point relationship between *in vitro* dissolution and the *in vivo* input rate (e.g., the *in vivo* dissolution of the drug from the dosage form). In a linear correlation, the *in vitro* dissolution and *in vivo* input curves may be directly super-imposable or may be made to be super-imposable by the use of a scaling factor” [10]. For example, if the dissolution is faster than the in vivo input rate then the two curves are not super-imposable. In this case a time scaling may be applied on the *in vitro* data in order to find, for each percentage absorbed, the corresponding time *in vitro*. This is usually carried out using a Levy's plot.

Model predictability was estimated internally by comparison of prediction errors on pharmacokinetic parameters used to establish bioavailability (BA) and bioequivalence (BE): Cmax, Tmax and AUC, derived from mean observed and predicted *in vivo* data obtained by convolution method. For a reasonable IVIVC, regulatory guidelines state prediction errors for Cmax and AUC should not exceed 10 % as a mean and none greater than 15% [10,11].

All calculations were done using Microsoft Excel. As the purpose of this paper is to demonstrate the feasibility to use IVIVC for the current tablets, all the calculations were performed on mean curves calculated based on all subjects (mean plasma time-concentration and mean of absorption kinetics) and on mean of the dissolution.

## 3. Results and Discussion

### 3.1. Results

**Figure 2.**
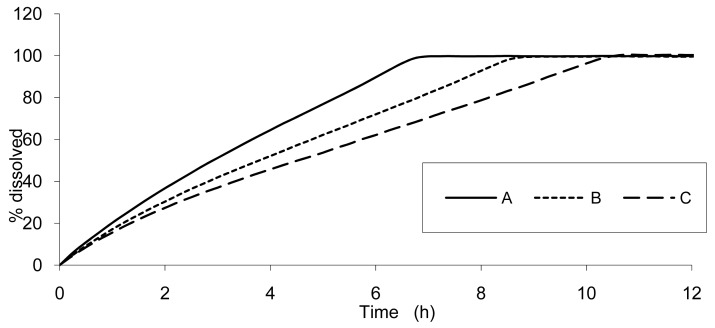
*In vitro* dissolution of the test formulations **A**: 6 mm, **B**: 7.5 mm, and **C**: 9 mm in USP2 pH 6.8 phosphate buffer, 50 rpm. The relative standard deviation is below 4 % at all time points. The test formulations are fully dissolved (100%) after approximately 6.5, 8.5 and 10 hours, respectively.

**Figure 3.**
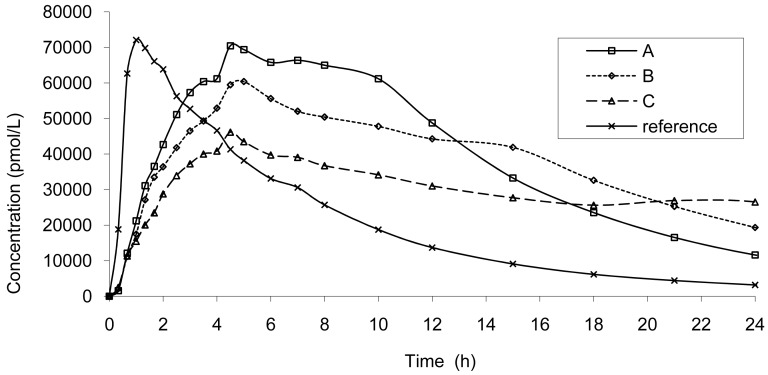
Plasma concentration up to 24 hours post dose of the three test formulations (**A**, **B** and **C**: test tablet formulations, dose = 20 mg) and of the reference tablet (IR, dose = 10 mg)

The result of dissolution analysis of the three test formulations is presented in Figure 2 and the plasma concentrations obtained by *in vivo* studies are presented in Figure 3.

**Figure 4.**
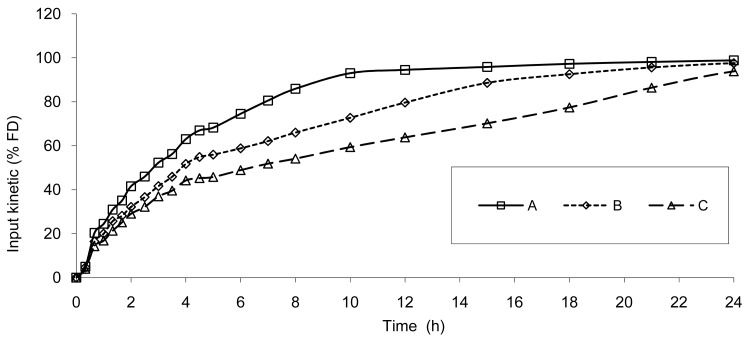
Input kinetics of Hydrocodone after administration of the 3 test tablet formulations based on deconvolution using IR data, **A**: 6 mm, **B**: 7.5 mm and **C**: 9 mm.

Based on the IR formulation, the deconvolution was assessed and provided the results presented in Figure 4. The curves represent the *in vivo* release of Hydrocodone of the three test tablets; these kinetics are called “input” kinetics in this paper.

The bioequivalence results on AUC and Cmax are presented in Table 2.

The analysis of the data indicated that for the AUC, the ratio between the IR and PR tablets corrected by the dose were of 1.00, 1.0. and 0.93 for tablet formulations A, B and C respectively, and the coefficient of variation (CV) of the analysis of variance (ANOVA) was 7.4%. For Cmax, the ratios corrected by the dose were 0.49, 0.40 and 0.30 for tablet formulations A, B and C, respectively, and the CV of the ANOVA was 14%. The results indicate that the AUC of all formulations are bioequivalent but a difference could be observed on Cmax. The coefficients of variations, which were good estimates of the intra subject variability, were low, indicating that the IVIVC could be used to predict minimal variation between tablet formulations with a good discriminative power.

**Figure 5.**
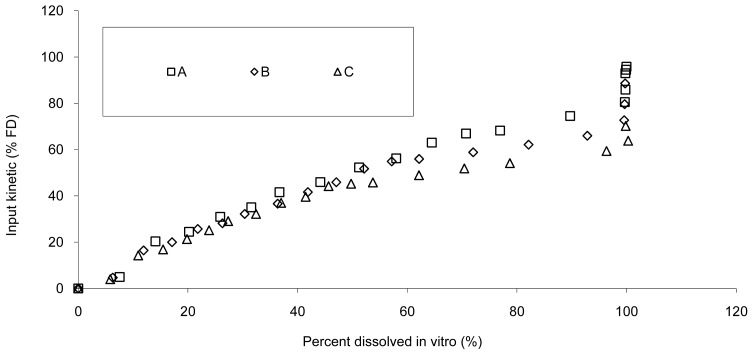
Dissolution *vs*. input kinetics of Hydrocodone after administration of tablet **A**, **B** and **C**, first attempt of IVIVC.

All tablet formulations were used to establish IVIVC; the resulting *in vivo* absorption as a function of *in vitro* release is presented in Figure 5.

**Figure 6.**
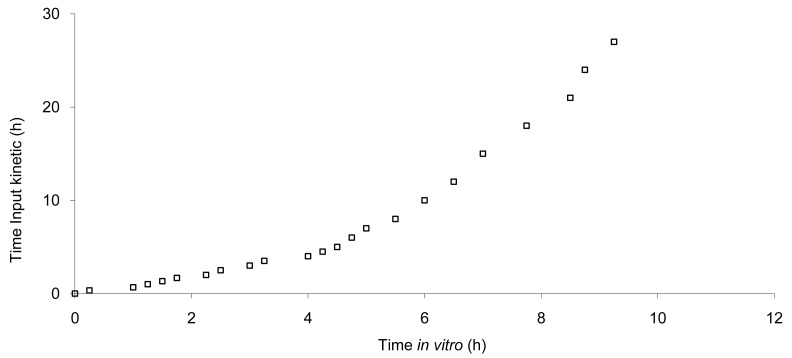
Levy's plot for time scaling.

**Figure 7.**
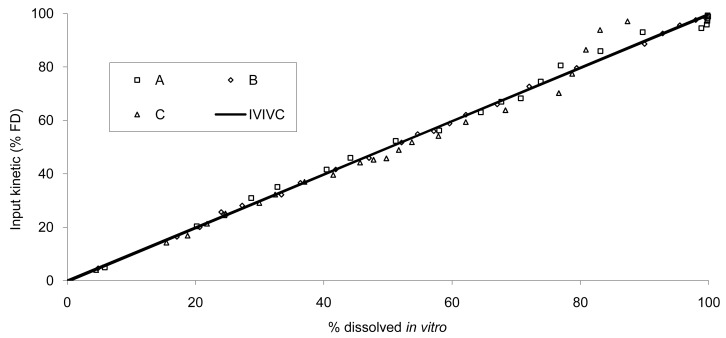
IVIVC established on all formulations using a non-linear time scaling common to all formulations.

The data show that a small lag time exists *in vivo* and not *in vitro*. In addition, the dissolution is too fast after four hours compared to the *in vivo* release of the tablet; for example 100% dissolved leading to only 81% absorbed. The *in vitro* dissolution and *in vivo* input curves were not directly super-imposable and were made super-imposable by the use of a scaling factor as described in FDA note for guidance [10]. A time scaling was therefore employed leading to corresponding earlier dissolution time *in vitro* coupled with relatively later *in vivo* input results. In practice, the time observed *in vitro* to fit to each percent of fraction of dose absorbed *in vivo* were searched (Figure 6) and a common time scaling factor for all formulations was established. This time scaling is non-linear denoting that processes explored *in vivo* and *in vitro* were not consistently similar, the *in vivo* process was slower when time increased compared to *in vitro* (this point is discussed in Section 3.3). The resulting IVIVC is presented in Figure 7.

**Figure 8.**
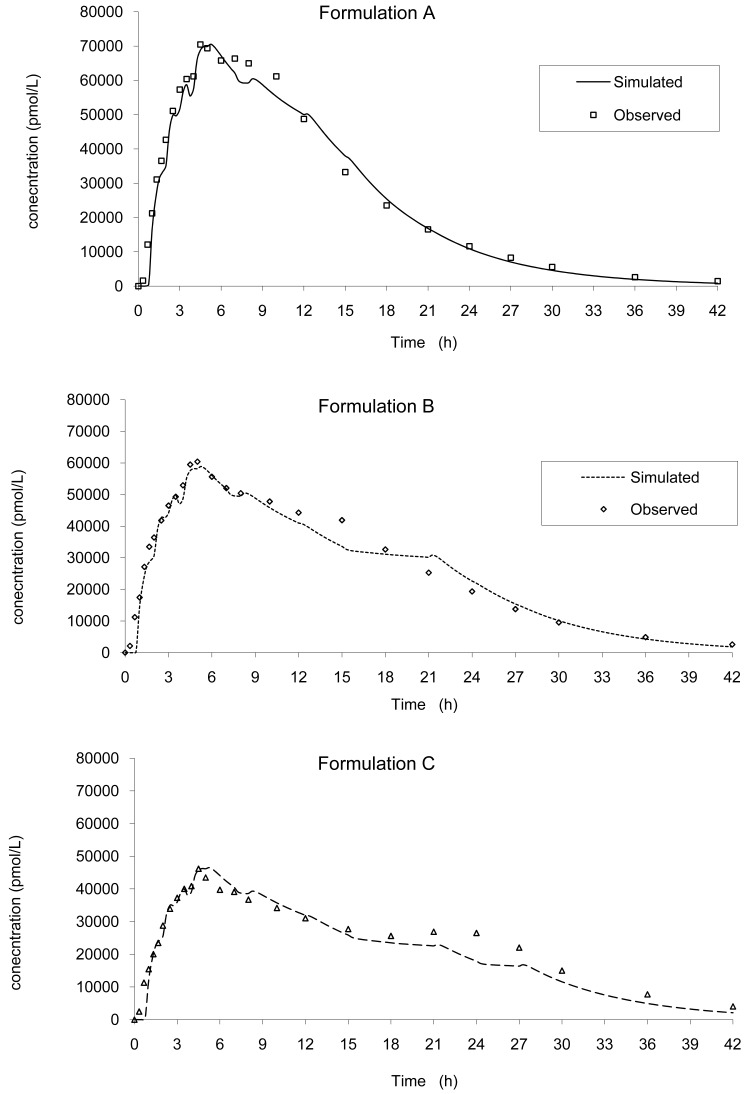
Simulated mean curves obtained based on IVIVC (lines) *vs*. observed mean values (square = formulation **A**, diamond = formulation **B** and triangle = formulation **C**): internal predictibility.

Based on this IVIVC and on *in vitro* data, the input kinetics were back calculated and then, based on this input function, a convolution was performed to simulate the *in vivo* plasma concentration curve without any correction of the dose (the labeled 20 mg was used). Based on the simulated values, the mean PK parameters were calculated and compared to the original one. The results are presented in Figure 8 and Table 3 for all tablets.

The predictability was good and in accordance with the FDA recommendation (5) with a mean error of −0.32% and −6.63% on Cmax and AUCinf, respectively, no case being greater than + or −10%.

### 3.2. Discussion on results

A common challenge of all pharmaceutical companies is to develop new products as fast as possible to cover unmet medical needs, and to ensure, at the same time, safety and efficacy. Many strategies exist and amongst them, *in vitro* dissolution and *in vitro*–*in vivo* correlation (IVIVC) can be used early in the development phase in order address impact of changes in dosage forms with regard to bioequivalence (BE) and to improve the development of them so they reach the market faster. This option was chosen to address: (i) the high concentration (Cmax) observed with the 10 mg IR tablet without impairing the global exposure (AUC); and (ii) to investigate the possibility of a once a day tablet maintaining if possible a higher concentration at 24 h, in contrast with the current IR tablet which requires at least a two time a day administration and exposes subject to high Cmax and possibly low level before the next intake, leading to incomplete pain relief and management. Prototype tablets of prolonged release 20 mg tablets were developed in order to sustain concentrations to be able to have a once a day tablet: to obtain an AUC doubled *vs*. 10 mg, a longer Tmax and lower Cmax, comparable to the IR tablet. This first goal was achieved as the AUC were doubled for all test tablets and the Cmax corresponded at maximum to the 10 mg IR tablet.

The IVIVC based on the current study could be used as the predictability was correct: (i) to optimize the tablet formulation; but also (ii) to help for regulatory purposes, like fixing dissolution limits, scale up and post approval changes. The findings are coherent with the biopharmaceutical class of Hydrocodone—Class 1 according to Biopharmaceutical Classification System BCS (high solubility, high permeability, [5,9])—as the release from the drug dosage form is the only limiting factor and neither the solubility of the active pharmaceutical ingredient nor the absorption. AUC was not a parameter anticipated to cause problems, as the drug, upon release, is absorbed. The main prerequisite is simply that the IVIVC predict accurately if all parameters implied in the release and the release mechanism remain constant. Based on the current IVIVC, any tablet formulation between formulations A and C (6 to 9 mm) could be predicted and any modification of the dissolution curve be simulated *in vivo* to estimate the impact on bioavailability and possibly on *in vivo* efficacy.

### 3.3. Calculation of dissolution limits

One application of IVIVC is to predict bioavailability and to set dissolution limits. Based on the result of the BE study for the main parameters of interest, Cmax and AUC up to infinity, the residual error variance was extracted from the ANOVA and estimated to be 14 and 7.4% respectively (coefficient of variation). Using this value, in formulation B for example, the limits to have Cmax and AUC within the bioequivalence limits were estimated by Equations 1 and 2.

Eq 1: $$e^{\left[\text{Ln}(0.80)+\frac{t\times s_{r}}{\sqrt{n}/2}+\text{Ln}({\bar{m}}_{\text{ref}})\right]}={\bar{m}}_{\text{lower}}$$

Eq 2: $$e^{\left[\text{Ln}(1.25)–\frac{t\times s_{r}}{\sqrt{n/2}}+\text{Ln}({\bar{m}}_{\text{ref}})\right]}={\bar{m}}_{\text{higher}}$$

**Figure 9.**
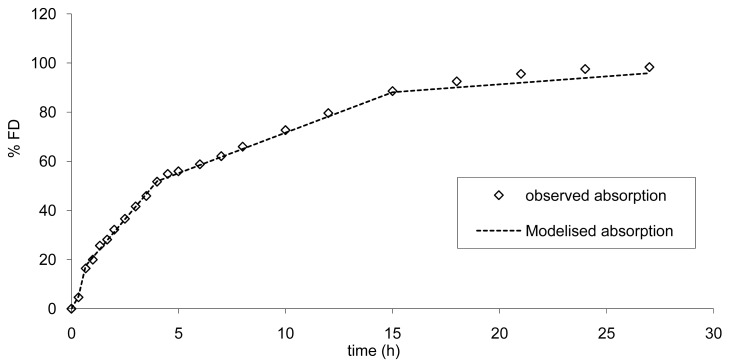
Modelization of a multi zero order absorption according to Equation 3.

To establish the limits, the absorption was modelized according to a multi zero order absorption (Equations 3.1 to 3.3) and the results are presented in Figure 9.

Eq 3.1 $$t\in ]0.33-4]\Rightarrow {\text{Abs}}_{1}(t)=100-[89.4–10.32\times t]$$

Eq 3.2 $$t\in ]4-11]\Rightarrow {\text{Abs}}_{2}(t)=100-\left[{\text{Abs}}_{1}(t_{4})-3.29\times (t-t_{4})\right]$$

Eq 3.3 $$t\in ]11-\text{end}]\Rightarrow {\text{Abs}}_{3}(t)=100-\left[\text{Abs}2(t_{11})-0.65\times (t-t_{11})\right]$$

This modelization is necessary in order to calculate the increase or decrease in absorption rate resulting in a modification of AUC and Cmax within the limits of bioequivalence. A similar mechanism of absorption was used. Based on the results of the modelization of the absorption the mean error from the model was of −0.97% confirming the good accuracy of the model. These findings are in accordance with a scintigraphic study published employing an Egalet® matrix system loaded with caffeine [16]. The presence of the formulation was monitored through the small intestine, ascendant and transverse colon, and descendent colon. Rate of absorption was estimated to decrease gradually in each part of the intestine. This modelization of absorption could directly be linked to the observed plasma curves (Figure 3 and Figure 8) and the GI transit. A shoulder was observed around 4 h on the mean plasma curves and could be linked to the transit from the small to the large intestine. In addition, the non-linear time scaling, as described in Figure 6, could also be explained by where the tablet was in the GI tract and the resulting absorption rates.

Those findings confirmed the observed difference in release rate between *in vivo* and *in vitro* data which was estimated to be around 4 hours and could be linked with the passage of the tablet from the small intestine to the colon. The release mechanism *in vivo* was not reflected in a similar magnitude *in vitro*, where constant conditions were applied along the 10 hour period, explaining the need for time scaling.

**Figure 10.**
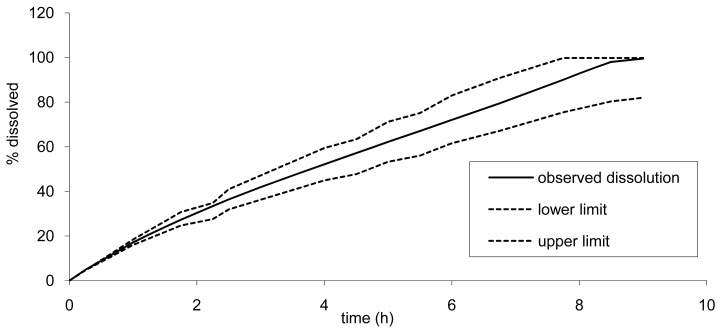
Dissolution limits to ensure bioequivalence to formulation B.

However, as the API cannot be absorbed more than 100%, the upper limit would reach 100% earlier. The lower limits for Cmax and AUC were of importance here to determine the lower dissolution limits. This slower dissolution would impact the rate of absorption but also the quantity released and absorbed as a part of the drug could be expelled in the feces and not absorbed as the release would be too slow. In our simulation, we fixed arbitrarily the maximum transit time and release limits to 24 h. The impact of a longer transit time (36 h) was investigated and led only to a marginal increase of AUC of less than 5% for the lower limit (slow tablet). The dissolution limits calculated based on this principle are presented in Figure 10.

**Figure 11.**
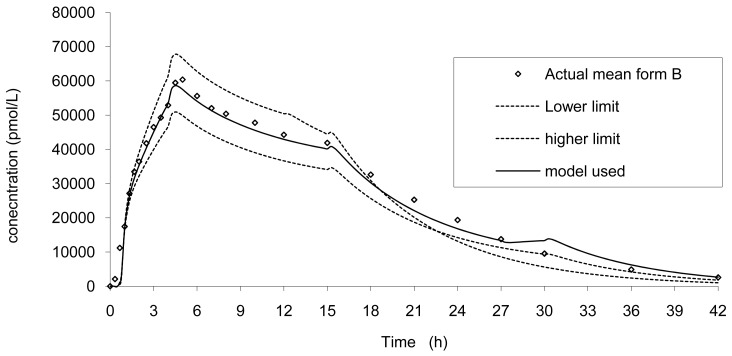
*In vivo* prediction of formulation B based on the lower and upper dissolution limits to ensure bioequivalence.

Based on the dissolution limits and on the modelization of the absorption, the *in vivo* curves were simulated in three conditions corresponding to the modelized absorption. The results are presented in Figure 11.

**Figure 12.**
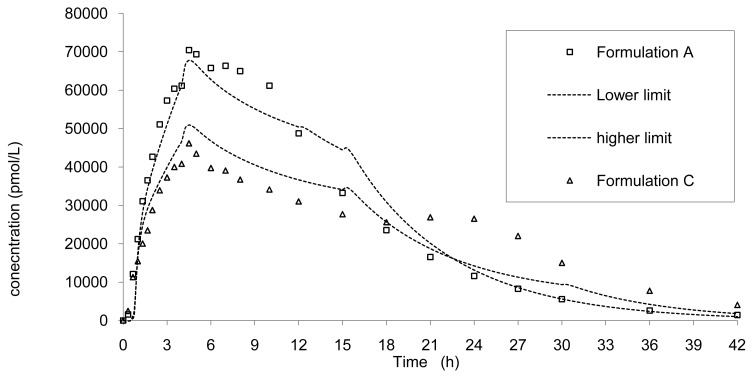
*In vivo* formulations A and C and the lower and upper limits based on calculated dissolution limits of formulation B to ensure bioequivalence.

The rapid release formulation A and the slower C are presented in Figure 12 associated with the previous limits.

It can be observed that these two formulations A and C, corresponding to a faster and slower release, did not fall within the limits calculated based on formulation B results that confirm the absence of bioequivalence observed for Cmax (Table 2).

### 3.4. Calculation of optimized formulation

Based on the results from the BE study, the dissolution limits to ensure BE of the Cmax and AUC of any tablet formulation *vs*. tablet B was calculated using the IVIVC. Thus all formulations would be bioequivalent with the formulation B (7.5 mm) if the release rate estimated by dissolution is within the limits proposed in Figure 10. For example, based on the IVIVC, an *in vitro* dissolution, optimizing the profile leading to similar AUC but more prolonged and decreased Cmax, could be estimated. Figure 12 presents the target dissolution profile compared to the existing one and to the limits in which bioequivalence is ascertained.

**Figure 13.**
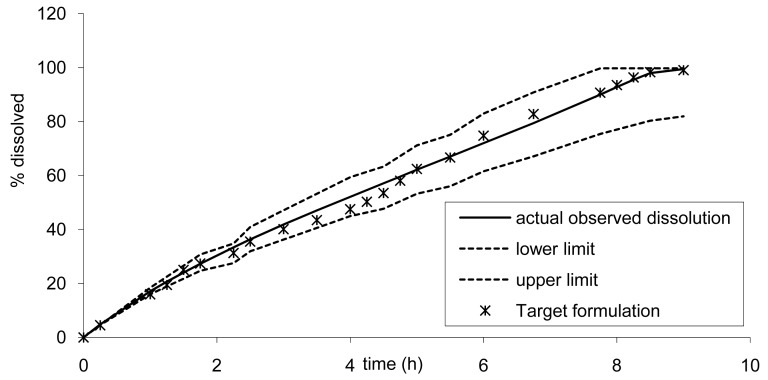
Dissolution on a target formulation derived from formulation B to ensure decrease of Cmax maintains a plateau.

Based on the dissolution, it could be anticipated that new tablet formulations would be bioequivalent with the existing formula. The calculation of the 90% confidence interval (CI) gave the following results 0.89–0.97 and 1.01–1.06 for Cmax and AUC infinity, respectively. Thus the development of this new tablet formulation could simply have a definite target dissolution profile for the formulators, at a very low risk of BE study failure. Alternatively, the developed tablet could even be submitted as a new drug application based on a surrogate dissolution test only, as presented in Figure 13 [10,11].

Another example on the possible usage of these limits is for use in the submission dossier, in accordance with the FDA note for guidance, for the scale up with a scaling factor greater than 10. As dissolution limits were calculated and ensure bioequivalence on the main PK parameters, if the dissolution observed in the full scale batch would be within the limits, no BE study would have to be carried out to support the scaling up. Similarly, any modification in the tablet within a predefined range for the excipients could be supported solely by dissolution as a BE surrogate.

A similar exercise could be performed for formulation A and C and for any formulations in between as a similar release mechanism and time scaling were observed for all three formulations.

## 4. Conclusions

The work presented in this paper emphasizes the importance of dissolution as a prediction technique for development and optimization of tablet formulation, but also as a quality control tool. The IVIVC was initiated and validated for three formulations. A good internal predictability was observed for all tablet formulations. The validated IVIVC could be used to optimize the formulation and to achieve a desired profile. In addition, this technique could help to establish the dissolution limits associated with a certainty of bioequivalence. Based on this validated level A, IVIVC dissolution could be used as surrogate of bioequivalence.

## Figures and Tables

**Table 1.** Main formulation characteristics.

**Formulation**	**A**	**B**	**C**
Strength mg	20	20	20
Length mm	6	7.5	9
Short diameter mm	4.7	4.4	4.4
Long diameter mm	9.4	8.6	8.3
Average tablet mass mg	240	257	314

**Table 2.** Result of bioequivalence study (corrected by the dose).

	**Comparator**		**Cmax**	**AUC inf**
A 6 mm	Reference	Ratio	0.49	1.00
		90% CI	0.45–0.52	0.96–1.04
B 7.5 mm		Ratio	0.40	1.03
		90% CI	0.37–0.43	0.99–1.07
C 9 mm		Ratio	0.30	0.93
		90% CI	0.28–0.32	0.89–0.97
A 6 mm	B	Ratio	1.20	0.97
		90% CI	1.12–1.30	0.93–1.01
C 9 mm		Ratio	0.75	0.90
		90% CI	0.70–0.81	0.87–0.94

**Table 3.** Predictability based on AUC and Cmax of the mean curve.

**Formulation**	**Simulated on mean data**			**Observed on mean data**			**Deviation**		
	Cmax pmol/L	AUC 0-42 pmol.h/L	AUC inf pmol.h/L	Cmax pmol/L	AUC 0-42 pmol.h/L	AUC inf pmol.h/L	Cmax %	AUC 0-42 %	AUC inf %
A	70332	1016005	1021836	70449	1052679	1064817	-0.17	-3.48	-4.04
B	56421	1007388	1019908	60393	1083974	1107549	-6.58	-7.07	-7.91
C	48839	902842	918345	46166	960346	997534	5.79	-5.99	-7.94
Mean predictability							-0.32	-5.51	-6.63
